# MYCbase: a database of functional sites and biochemical properties of Myc in both normal and cancer cells

**DOI:** 10.1186/s12859-017-1652-6

**Published:** 2017-04-28

**Authors:** Debangana Chakravorty, Tanmoy Jana, Sukhen Das Mandal, Anuradha Seth, Anubrata Bhattacharya, Sudipto Saha

**Affiliations:** 1 Bioinformatics Centre, Bose Institute, P 1/12, C.I.T. Road, Scheme-VII (M), Kolkata, 700054 India; 2 St.Xavier’s College, Kolkata, India; 3 Biochemistry Department, University of Calcutta, Kolkata, India; 4 Current Address: Department of Biological sciences, Indian Institute of Science Education and Research, Kolkata, India

**Keywords:** Myc, Cancer, Mutations, Protein-protein interactions, Metabolism

## Abstract

**Background:**

Myc is an essential gene having multiple functions such as in cell growth, differentiation, apoptosis, genomic stability, angiogenesis, and disease biology. A large number of researchers dedicated to Myc biology are generating a substantial amount of data in normal and cancer cells/tissues including Burkitt’s lymphoma and ovarian cancer.

**Results:**

MYCbase (http://bicresources.jcbose.ac.in/ssaha4/mycbase) is a collection of experimentally supported functional sites in Myc that can influence the biological cellular processes. The functional sites were compiled according to their role which includes mutation, methylation pattern, post-translational modifications, protein-protein interactions (PPIs), and DNA interactions. In addition, biochemical properties of Myc are also compiled, which includes metabolism/pathway, protein abundance, and modulators of protein-protein interactions. The OMICS data related to Myc- like gene expression, proteomics expression using mass-spectrometry and miRNAs targeting Myc were also compiled in MYCbase. The mutation and pathway data from the MYCbase were analyzed to look at the patterns and distributions across different diseases. There were few proteins/genes found common in Myc-protein interactions and Myc-DNA binding, and these can play a significant role in transcriptional feedback loops.

**Conclusion:**

In this report, we present a comprehensive integration of relevant information regarding Myc in the form of MYCbase. The data compiled in MYCbase provides a reliable data resource for functional sites at the residue level and biochemical properties of Myc in various cancers.

## Background

Myc is a multifunctional protein and is believed to regulate expression of 15% of all genes through binding on Enhancer Box sequences (E-boxes) CACGTG and recruiting histone acetyltransferases (HATs) [1]. It has a major role to play in the cell cycle, cell growth, differentiation, apoptosis, transformation, genomic stability and angiogenesis [2]. Since it is a key regulator of all essential activities in the cell, its over-expression is responsible for causing different types of cancers including ovarian cancer [3] and hepatocellular carcinoma [4]. The role of Myc in the development of Burkitt’s lymphoma has been well documented. In many human malignant tumors, the overexpression of Myc by gene amplification, proviral insertions, as well as chromosomal translocation has been observed [5]. Along with these types of mutations, non-synonymous point mutations have also been widely reported in the scientific literature [6]. These mutations result in deregulation of genes involved in cell proliferation as most genes under the control of Myc are vital for survival [7]. c-Myc protein is composed of an N-terminal domain (NTD), a C-Terminal domain (CTD) and a central region which is intrinsically disordered. The NTD, consisting of short motifs called the Myc boxes, for example, Myc box I (MbI) and Myc box II (MbII), is involved in most of the known Protein-protein Interactions (PPIs) [8]. On the other hand, the CTD, composed of the basic helix-loop-helix and leucine zipper region (bHLH-LZ), is involved in interaction with Max to form Myc-Max heterodimers [9]. Myc-Max dimer is responsible for transactivation of many genes which lead to proliferation and cancer [10]. The central region, being disordered, may be looked into to find short sequence motifs important in mediating PPIs [11].

### Other relevant information

With the advent of new technologies such as high throughput proteomics and genomics and development of epigenetics, much new information about Myc has been generated. The methylation pattern of the c-Myc gene has also been linked to colorectal cancer [12] which gives an insight into epigenetic regulation. Myc is stabilized by post-translational modification (PTM), most importantly by phosphorylation at Serine 62 (S62) [13]. This event primes c-Myc for the second phosphorylation at Threonine 58 (T58) which enhances its degradation through recruitment of FBXW7. The role of PTMs, especially phosphorylation and ubiquitination, in the regulation of biological processes mediated by Myc has been of interest to the scientific community for several years. Understanding the PTMs may, therefore, open new avenues into targeting c-Myc for inhibition of cell proliferation. Myc protein also regulates genes involved in several pathways including lipogenesis, glycolysis, glucose and glutamate import, lactate export, nucleotide biosynthesis and glutaminolysis [14]. Since Myc is involved in 70% of the cancers occurring in humans, it has the potential of becoming a drug target. In fact, many small chemicals targeting Myc-Max dimers have been designed to prevent transcription of proliferation-related genes [15]. Also, over-expression of Myc has been blocked by using some anti-G quadruplex compounds and BET inhibitors [16]. Other strategies that have been developed to target Myc are with the use of miRNAs such as miR-26a used in animal models [17].

### Need for the database

Over the last few decades, a substantial number of publications and conferences have been dedicated to specific key essential genes including p53 and Myc. Although an extensive amount of research is being done in the field of Myc biology, a gap remains in understanding and targeting the deregulated Myc in the disease state. The primary concern with anti-Myc therapy lies in the fact that it continues to be essential for normal proliferating tissues. Myc is also involved in a vast network of protein-protein interactions (PPIs) as well as protein-DNA interactions and modulates many signalling pathways which make it even harder to inhibit without causing serious side effects. A deeper understanding of these aspects of Myc may guide researchers into effectively targeting Myc for anti-cancer therapy. The foremost challenge lies in collecting the extensive amount of information from the vast reserve of scientific literature and secondary databases. Even though existing databases such as UniProt [18] and GeneCards [19] do provide information on various aspects of Myc such as gene sequence, size, an overview of interactors and PTMs, they lack precise details such as mutated residues and their link with PTMs or the region of Myc involved in PPI. These can only be obtained after the meticulous search of the scientific literature or browsing dedicated databases. It is therefore of utmost importance to accumulate all relevant data in a user-friendly platform in the form of a database from where the user may achieve new insights into disease biology and treatment.

In order to get an overall idea about the functional sites of Myc along with its properties, an attempt has been made to construct a database named MYCbase. MYCbase can be seen as a repository of all aspects of Myc relevant to its biochemical characterization including mutations, PPIs, small chemical drug molecule targeting, metabolic pathways, methylation pattern and others. We also focus on various analysis and conclusions that can be derived from this database which may help the scientific community in designing and troubleshooting their experiments in the field of Myc biology. To the best of our knowledge, there is no existing database, which compiles this variety and quantity of information regarding Myc, with relevance to cancer, studies under a single platform.

## Construction and content

### Database schema and implementation

MYCbase was developed as a relational database using the Apache HTTP 2.2.15 web server and MySQL 5.1.69. The PHP 5.3.3, HTML, JavaScript and CSS were used at the front-end of the database. The PHP-based web interfaces were designed to execute the SQL queries dynamically.

**Fig. 1:**
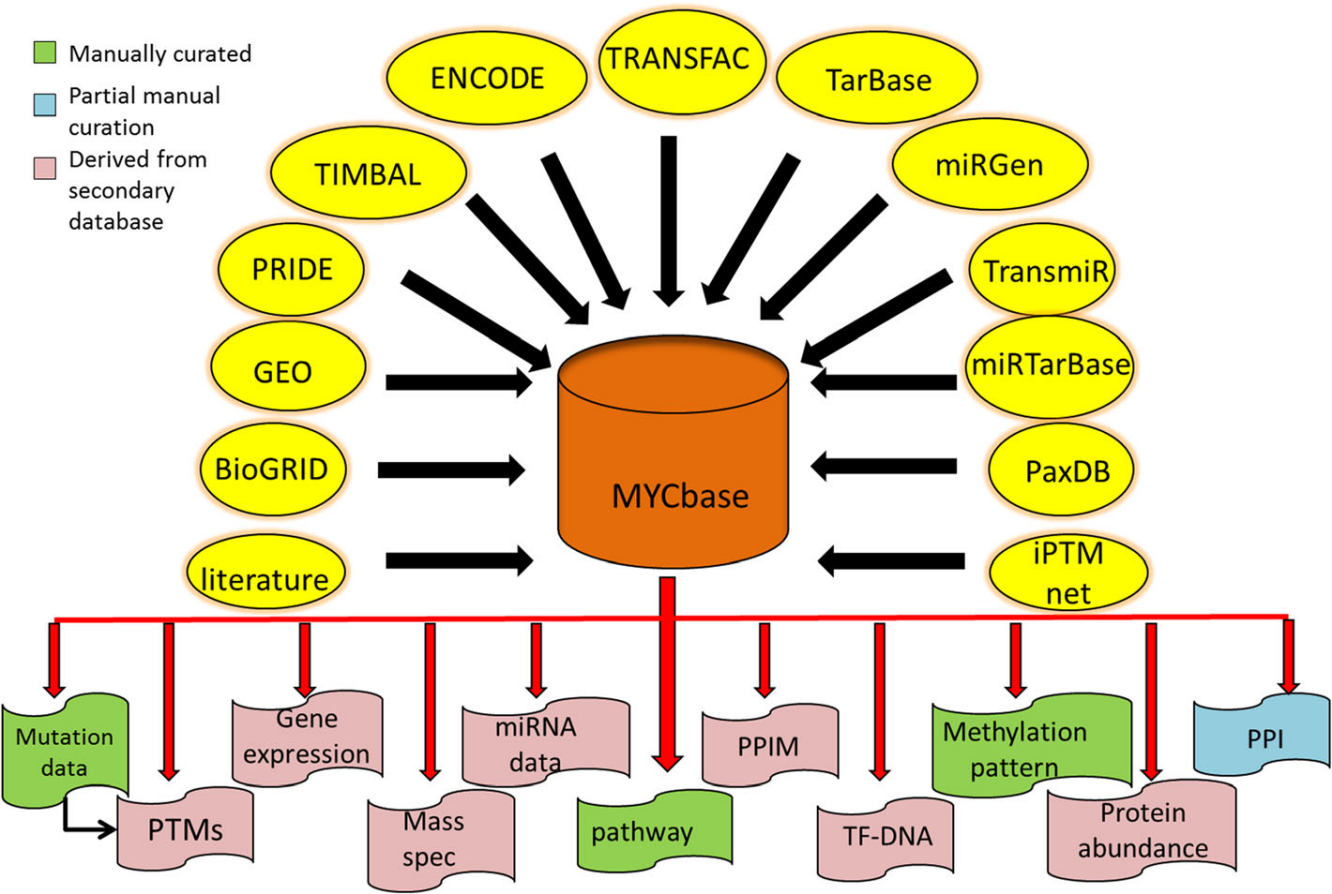
Schematic diagram of MYCbase architecture. A schematic representation of the source of the data and the categories of data contained in MYCbase

MYCbase comprises eleven categories of information about Myc protein and gene, collected from different resources as shown in Fig. 1. It is a tertiary database containing 2223 entries from various aspects of MYC. The data compiled can be divided into three broad categories. First, the manually curated data from PubMed literature, such as mutation (352), metabolism and pathway (126) and methylation pattern (29). Second, the partially curated data like protein-protein interactions (PPIs) (925), where the cell lines used in all the experiments, the region of Myc with which they interact and the outcome of the interactions were manually curated from scientific literature and added to the existing information derived from a specialized database. Third, the data derived from other specialized databases, including gene expression information of Myc (115), Mass-spectrometry related data of Myc protein (41), miRNAs interacting with c-Myc gene along with MYCN and MYC-Max (202), modulators of protein-protein interactions or PPIMs (30), DNA interactions of Myc (131), Myc protein abundance data in various cell lines and tissues (168) and finally post-translational modifications present in Myc (104).

### Data sources

#### Manually curated tables

**Mutation:** Around 446 different abstracts were downloaded from PubMed till November 2015 by using the keywords 'c-myc gene' and 'c-myc gene mutations'. All relevant publications were sorted to extract information on c-myc gene mutations. The manually extracted information was placed in categories such as the location of mutation, residue mutated, type of mutation, cell line or sample used, methods, the disease affected, follow-up method, PubMed ID, and frequency of mutations. The functional sites mutated at the residue level are also linked to the PTM table so that any modifications at the site of mutations can also be seen easily.

**Methylation pattern:** PubMed was searched for (Myc[Title]) AND (Methylation[Title]) from which 42 relevant hits were obtained till April 2016. Only 29 publications had relevant information about c-myc methylation with disease states. These findings were arranged under the region explored, the nucleotide sequence, methylation status, cell line used, an outcome of methylation and relation to any disease.

**Pathways/Metabolism:** PubMed was searched for Myc in connection with metabolism and pathway which gave 126 till November 2015 results with information pertinent to the database. All papers were thoroughly examined and results were grouped under pathway, enzyme, regulatory action, comments, cell line and PubMed ID.

#### Partially curated tables

**Protein-Protein Interactions (PPIs):** The already existing database for PPIs Biological General Repository of Interaction Datasets (BioGRID) [20] was searched to collect the PPIs of Myc protein which contained information of 925 interactions. This information was arranged under protein names, type of interaction, UniProt ID of the interactor, experimental technique, PubMed ID, interaction domain, cell line used and the function. The cell line, interaction domain and result of the functions were manually extracted from original publications by comprehensive PubMed search. Some interactions, for an example, Myc-Max, have more than one entry (46), using different methods, cell lines, and references. The presence of many entries under the same interaction adds confidence as it has been reported by more than one group of researchers.

#### Derived from other specialized databases

**miRNA interaction:** miRNAs that interact with (or target) mRNA of the c-myc to regulate its gene expression were collected from different publically available databases such as TarBase v7.0, miRTarBase release 6.1, miRecords TransmiR and miRGen v3 [21–25]. These contain experimentally validated interaction which totals to 202 appropriate results including miRNAs which target MYCN and MYC-Max along with the ones targeting c-myc gene. They were grouped under miRNA name, the database used, tissue, cell line, methods used, PubMed ID, MiRBase Accession numbers, the gene they target and relevant comments.

**DNA interaction:** As c-Myc protein is a transcription factor with a DNA binding site it controls the expression of many genes. We have collected interaction between Myc as a transcription factor with other genes from TRANSFAC database [26]. These 109 results were categorized into the gene, location within the gene, binding site identifier, binding reaction, effect and quality score. The ChIP-seq data from ENCODE project [27] were also included in MYCbase.

**Protein- Protein Interaction Modulators (PPIM):** TIMBAL [28] database was used to collect information regarding the modulators of Myc protein. A total of 30 modulators were collected and arranged in a table having target PPI, chemical name, smile, complex description, target UniProt ID, PDB ID, assay description, assay type, confidence description and type of interaction columns. The chemicals detail information was collected and hyperlinked from PubChem database.

**Gene Expression:** Gene Expression Omnibus (GEO), NCBI [29] was used to identify 255 c-myc gene expression datasets. Information regarding the title of the dataset, GSE number, cell line, method used to find out the results, platform used for the experiment, disease-associated and PubMed ID of related publication were compiled.

**Mass Spectrometry-based proteomics data:** PRoteomics IDEntifications (PRIDE), EMBL-EBI [30] was used to extract all proteomics data where Myc was identified. This search using (P01106 accession number) revealed 44 hits. The results were assembled according to the title, project ID, cell lines used, type of experiment done, disease-associated and PubMed ID.

**Protein abundance:** Myc protein abundance data was derived from PaxDb: Protein Abundance Database [31] which resulted in around 168 hits. The search results were arranged according to the source of the protein, the method used, abundance, rank, interaction consistency score, coverage and PubMed ID.

**PTM(s):** All known PTMs of Myc along with their functional sites of modifications at the residue level and enzymes responsible were elucidated in MYCbase. There are 104 appropriate entries downloaded from iPTMnet [32]. Their original sources of the information along with the PubMed IDs were reported.

## Utility

### User interface

**Fig. 2:**
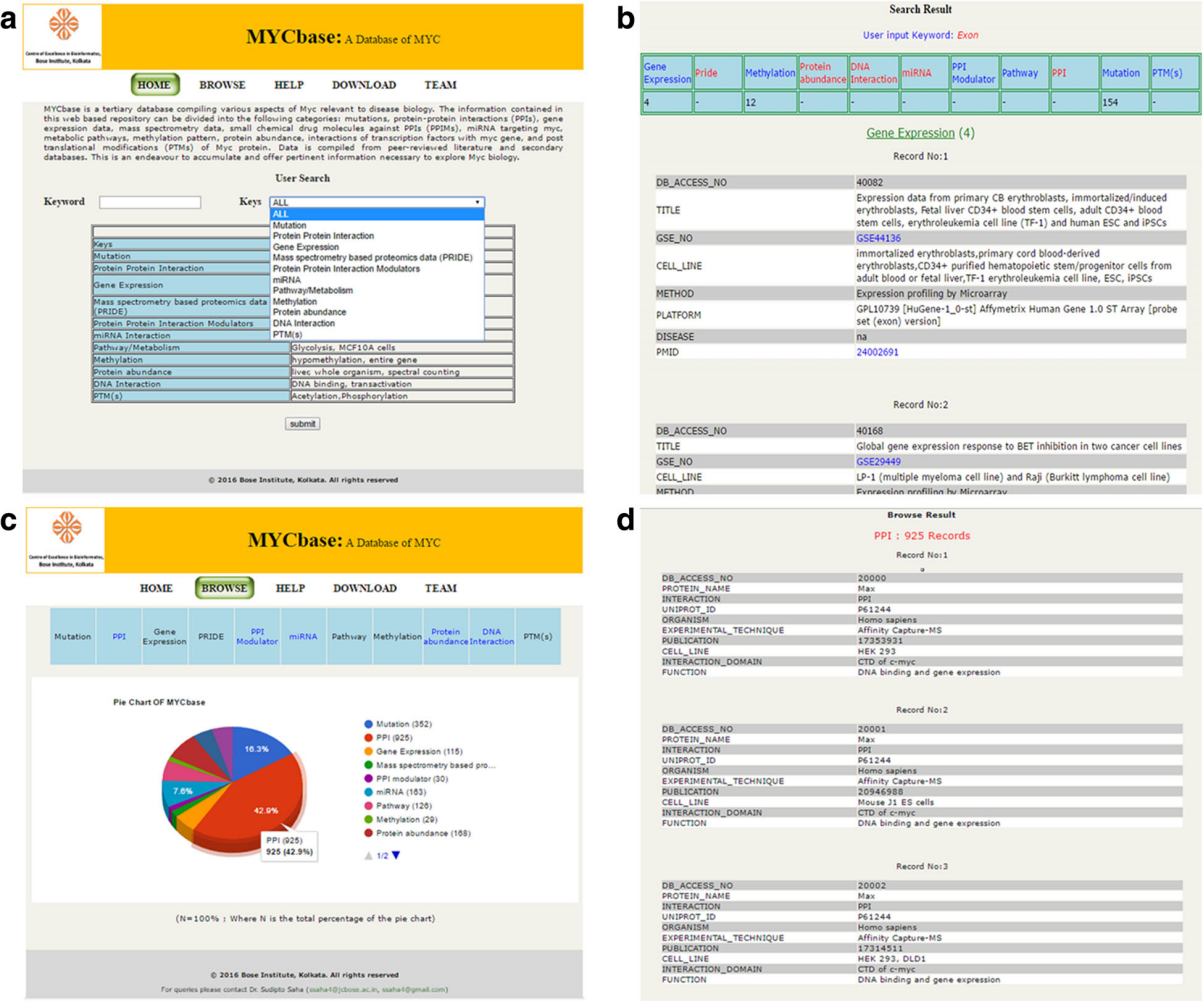
Screen shots of MYCbase (**a**) Search page (**b**) Output of search page (**c**) Browse page and (**d**) Output of browse page. **a**- In the search page a list of keywords for each set of keys is provided. The user can select any particular key (category) or use the ALL option to search for the keyword in all the categories included in MYCbase. **b**- The output for searching “Exon” in “ALL” categories is displayed here. The number of entries found in each category can be seen on top of the page and details can be seen as the user scrolls down. **c**- The browse page allows the user to browse through all entries in MYCbase. Hovering the mouse over each sector of the pie-chart gives the number of entries in that category. **d**- Once the “PPI” is selected the total number of entries is displayed and scrolling can access the entries

**Search Section:** The search option, available on the Home page, allows the user to search for one or multiple datasets using gene symbols, disease names, and tissue types. Users can either search using the ‘All’ option which gives results for all eleven categories or selects a category in which the keyword is to be searched. A list of keyword examples for each category is displayed on the home page as shown in Fig. 2a. The search will generate a table giving the number of entries matching the query in each of the eleven categories along with the list of all information matching the particular keywords in the database, as shown in Fig. 2b. The links to PubMed and other sites such as PubChem and UniProt, where ever applicable, is also given for further reference and understanding of the user.

**Browse Section:** MYCbase allows the user to browse the database and acquire information present in all the eleven categories. These are: i) Mutations, ii) Protein-Protein Interactions (PPI), iii) Gene expression, iv) Mass Spectrometry data (PRIDE), v) Protein-Protein Interaction Modulators (PPIM), vi) miRNA, vii) Pathway, viii) Methylation Pattern, ix) Protein abundance, x) DNA interaction, and xi) Post-translational modifications (PTMs). All information under each of these eleven categories can be accessed by clicking on them. In addition to this, a statistical representation in the form of a pie-chart is given to outline the percentage of data distribution under each category. Hovering the mouse over each sector of the pie-chart expands to show the number of entries under each category as shown in Fig. 2c and d.

### Other sections

Help Section: It provides an outline of the sources from which the data has been assembled. This section also gives the new user an idea of how to use MYCbase. Any queries related to MYCbase can be sent to the address mentioned on this page. Download Section: A dedicated download page helps the users to download all information present in the eleven categories available in MYCbase in .xls format. Team Section: The team page acknowledges the authors responsible for creating MYCbase.

### Data analysis

**Fig. 3:**
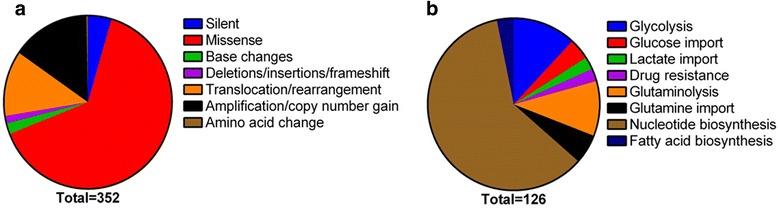
Distribution of MYCbase data from (**a**) type of mutations (*n* = 352) (**b**) different pathways regulated by Myc (*n* = 126). **a** Pie-chart of the types of mutations present under mutation category, **b** Pie-chart of the pathways in which Myc is involved in under Pathway category. Corresponding colour keys and a total number of data present are given for each pie-chart

**Fig. 4:**
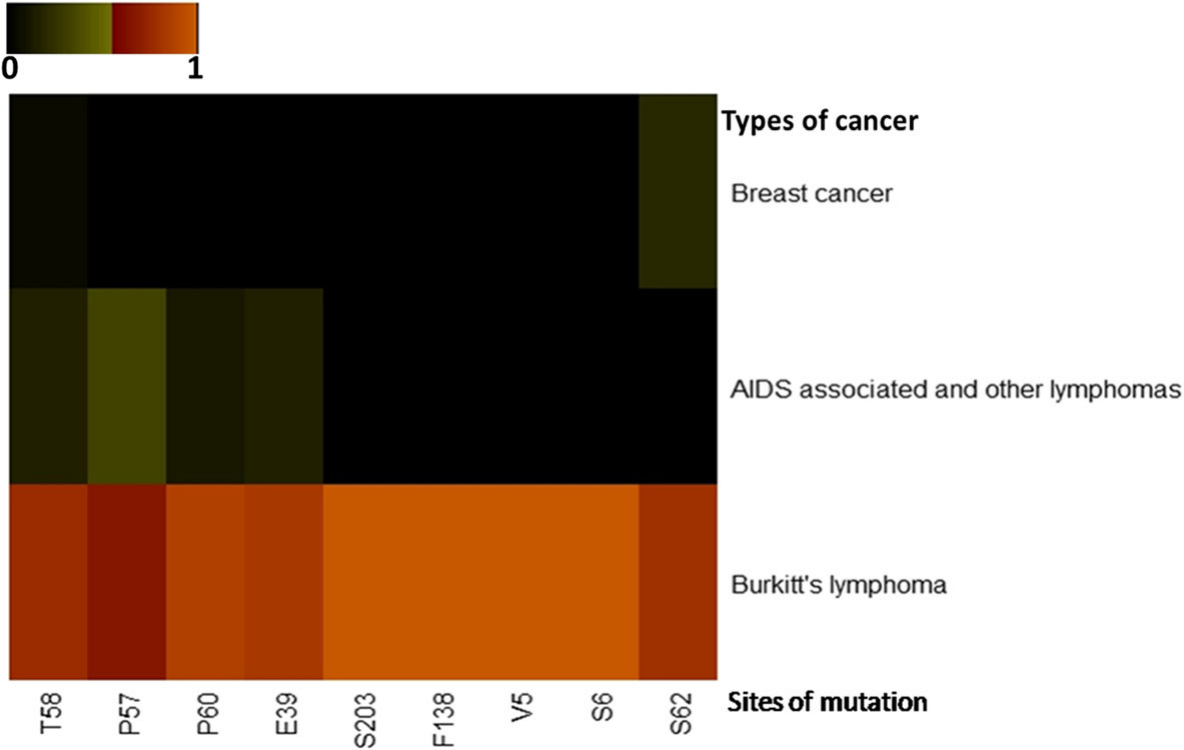
A heat map to display the trends of nine most frequently mutated residues of Myc gene (across 352 entries in MYCbase) against three different diseases (from MYCbase) caused by Myc mutations. For the heat map, the mutation proportion is represented in the form of a colour code given at the top of the figure. The values of mutation proportion range between 0 to 1.0, where 0 signifies no mutation in that residue for that disease and 1.0 signifies all the mutations for the residue are reported in the particular disease

The different kinds of mutations present in Myc gene can be represented in the form of a pie chart as shown in Fig. 3a. While translocation including the one present in Burkitt’s lymphoma remains to be one of the most documented mutations in the scientific literature, 43 out of 352 entries in MYCbase, some point mutations giving rise to other kinds of cancers were reported. From the 227 point mutations present in MYCbase the top most mutated residues were identified. Mutation proportions for the top nine most mutated residues were calculated by dividing the number of mutations at each site for the particular disease by the total number of mutations reported for that site. Top ten diseases caused by mutations present in the Myc gene were also derived from MYCbase (Table 1). A heat map was constructed using heatmap.3 function in R with default parameters to show the mutation proportions of nine most mutated residues for three diseases (Fig. 4). From this figure, we can conclude that mutations in S62 and T58 are present in all three diseases. Burkitt’s lymphoma, though being characterized by the most common translocation t (8; 14) (q24; q32), also shows mutations in all of the nine top mutated residues. The S62 and T58 being important sites for phosphorylation which is involved in proteasomal degradation of Myc may give a major clue as to why they are also most frequently mutated. For this reason, we have interlinked the sites of mutations and the sites of PTMs in MYCbase to give an idea as to how mutations in these regions may affect the regulation of Myc. Myc, as we see from MYCbase, plays an important role in regulating different cellular pathways (Fig. 3b). Myc is majorly seen to stimulate many genes involved in nucleotide synthesis, ribosome biogenesis, and translation. It is represented by 76 out of 126 entries in MYCbase under pathway/metabolism category. Myc is also seen to drive the expression of other genes that are involved in glucose import, glycolysis, amino acid uptake and catabolism, particularly of glutamine, supporting increased protein synthesis required for a growing cell.

**Table 1:** A list of top ten diseases associated with c-myc mutation as reported in MYCbase. The table comprises of top 10 different types of cancers present in MYCbase under mutations of myc gene and their corresponding entries for each type of mutations

Top 10 types of cancers	Total number of mutations	missense mutations	amplification	translocation	others
Burkitt's lymphoma	223	190	0	25	8
AIDS-associated and other lymphomas	41	24	0	1	16
breast cancer	13	2	7	3	1
gastric cancer including mucinous gastric carcinoma	8	2	4	0	2
lung cancer including small cell lung cancer	8	0	5	2	1
diffuse large B-cell lymphoma	5	0	2	3	0
acute lymphoblastic leukemia	5	0	0	5	0
multiple myeloma including plasma cell myeloma	5	0	0	5	0
plasma cell leukemia	4	0	1	3	0
gall bladder carcinoma	4	1	1	0	2

**Fig. 5:**
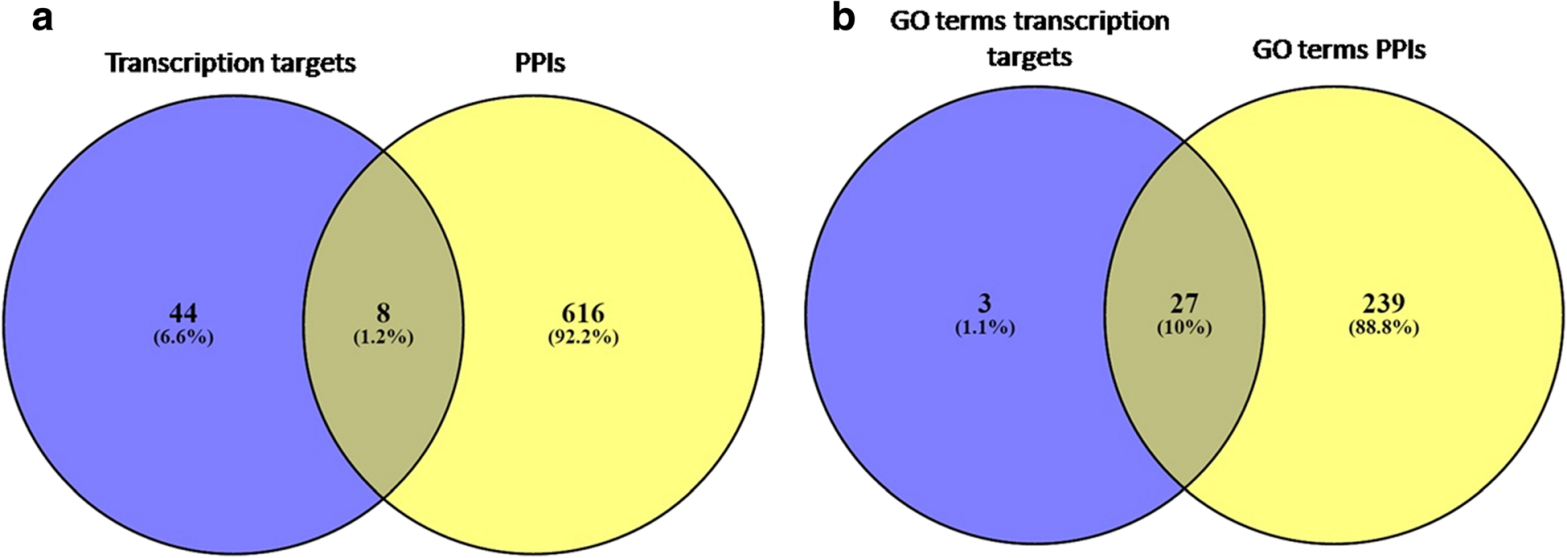
The Venn diagram shows the overlap of (**a**) between Myc transcription target genes and Myc PPIs and (**b**) between Myc Gene Ontology (GO) terms for transcription targets and PPIs. **a** A Venn diagram showing eight common targets which are transcribed by Myc as well as interact with Myc which may be involved in feedback mechanisms. **b** A Venn diagram showing 27 common GO terms associated with transcription targets and PPIs may give an idea of Myc-mediated regulation in different biological processes

The region of interaction of PPIs is a new addition to data already present in existing databases. From this, we get to know that little information is available for the central disordered region of c-Myc protein. It would be of interest to explore interaction motifs present in these regions which may be essential for PPIs. It was observed that few proteins which are Myc interactors in PPI table are also present as genes regulated by Myc in DNA interactions which are transcription targets of Myc. These target proteins can be speculated to be involved in transcriptional feedback loops. To identify these common targets we created a Venn diagram with the help of Venny 2.1 using data present in the two tables from MYCbase (Fig. 5a) [33]. We have identified eight proteins namely Bromodomain-containing protein 7 (BRD7), Cyclin-dependent kinase 4 (CDK4), Eukaryotic translation initiation factor 4E (EIF4E), Zinc finger protein GLI1 (GLI), Heat shock protein HSP 90α (HSP90AA1), Galectin-1 (LGALS1), DNA replication licensing factor MCM7 (MCM7) and S-phase kinase-associated protein 2 (SKP2) as the eight common targets. These targets are therefore involved in more than one type of relation with Myc and hence may prove to be involved in regulation of Myc and the coordination of its multiple functions. Most of these common targets are involved in cell cycle regulation and transcriptional activities. They can be further explored in future to establish the feedback loops. We also found out the Gene Ontology (GO) terms for each of the proteins under the PPI and DNA interaction categories using AmiGO 2 Term Enrichment Services for Biological processes [34]. The results from this were filtered such that any GO terms below level three and above level five were removed and analyzed using Venny 2.1 to find the common biological processes for the two molecular relations (Fig. 5b). We found 27 common GO terms for PPIs and DNA interactions. Some of the common GO terms were the regulation of cell cycle arrest (GO:0071156), cell cycle phase transition (GO:0044770) and regulation of cell proliferation (GO:0042127).

### A case study of breast cancer using MYCbase

We analyzed data regarding breast cancer in MYCbase by performing a simple keyword search with “Breast Cancer” in all options on the home page. The results we received, summarized the role of Myc in breast cancer. Gene amplification, including duplication, is the most important type of mutation that has been reported in breast cancer (in 7 out of 11 cases present in MYCbase). This is consistent with the knowledge that MYC amplification plays an important role in breast cancer development, progression and also associated with poor outcome [35, 36]. Breast cancer-associated gene, BRCA1, which functions as a tumour suppressor, binds to c-Myc protein and repress transcription is represented in the MYCbase PPI table with validated experimental techniques [37]. It is also represented in DNA interactions, as Myc is able to activate transcription of BRCA1 thus confirming that the loss of BRCA1 plays an important role in breast cancer [38]. Two missense mutations present in MYC found in breast cancer corresponds to S62 and T58, which, as already mentioned, are important sites for PTMs leading to proteasomal degradation of Myc. This study thus shows that MYCbase gives us a better representation of various aspects of Myc from a single platform.

## Discussion

The recent development in proteomic, transcriptomic and whole genome sequencing methodologies have led to the generation of extensive amounts of data and research articles related to c-Myc. MYCbase was developed by compiling the literature and existing secondary databases. MYCbase contains information of various functional sites at the residue level and biochemical aspects at a single platform, using which researchers can gain complete knowledge of the role of Myc in disease biology. MYCbase will be updated regularly to incorporate any updates in the secondary databases as well as in scientific literature.

Myc is a master regulator and can regulate of over 1500 coding and non-coding genes that include approximately 48 validated transcription factors [39, 40]. Many different methods have been used to identify these DNA, as well as, protein level interactions. For over a decade high throughput methods such as ChIP-seq and AP-MS are being used to provide a better understanding of Myc as a “hub” for these complex interactions. The data available in ENCODE project is the largest available repository for ChIP-seq data of human TFs and hence enriches MYCbase with high quality DNA-Myc interaction data. Myc may also be involved in feed forward loops which play a regulatory role both at transcriptional and post-transcriptional levels (miRNA mediated) [41]. MYCbase highlights the miRNAs that Myc, as a transcription factor, regulates (data from TransmiR) along with miRNAs that target MYC gene (data from miRGen v3). These miRNAs are of importance to regulate the expression of Myc protein, as well as, control downstream signalling processes. In addition, miRNAs which target MYCN and MYC-Max and have also been explored with miRGen v3 [25] and are included in MYCbase. ChIPBase is another database that incorporates ChIP-seq data which may be used to find out miRNAs that regulate Myc [42, 43]. The complex PPI network of Myc may be of interest to identify targets for anti-Myc therapy, which stands as a promising aspect for further studies by manipulating it experimentally. Till date, however, only PPIMs against Myc-Max and some bromodomain-containing proteins regulating Myc (BET inhibitors) have been identified [15, 16]. Therefore, there is the need to understand this complex Myc network to identify novel PPIs which can be targeted by small molecule inhibitors for proper regulation of Myc in disease states.

The data analysis of MYCbase has enabled us in drawing some conclusions on metabolic pathways and mutations at the residue level. First, Myc plays a major role in nucleotide biosynthesis which is essential for proliferating cells involved in tumorigenesis. A possible mechanism by which Myc regulates nucleotide biosynthesis is postulated to be through regulation of phosphoribosyl-pyrophosphate synthetase 2 (PRPS2) which results in promotion of increased nucleotide biosynthesis [44]. Second, Myc is also involved in glutamine synthesis and import pathways which are upregulated by overexpression of Myc to supply increased energy demands in a cancer cell [45]. Third, mutations present in Myc gene were studied for decades and particular emphasis is given to translocation of Myc gene present in Burkitt’s lymphoma. But, silent mutations present in Myc also have a major role to play in cancer. It was reported recently that silent mutations disrupt the interaction surface mediating PPIs [46]. This may open up a new front in the importance of exploring the regions of interaction involved in PPIs in Myc through the idea of “edgetic” perturbation [47]. Fourth, the mutations in T58 and S62 sites in the top three diseases caused mutations of Myc gene including Burkitt’s lymphoma are also important PTM sites. It has been seen that decreased T58 and increased S62 phosphorylation is present in human cancer cell lines associated with increased c-Myc protein stability [48]. Furthermore, the eight proteins which were identified as common targets both in Myc-protein interactions and Myc-DNA binding may be exploited in feedback regulations of Myc. Networks generated from such studies may help to elucidate at which level Myc can be regulated for controlling cancer progression.

## Conclusion

MYCbase is a tertiary database compiling various aspects of Myc relevant to cancer biology. Information present here has been accumulated from peer-reviewed literature and secondary databases. It is an open access database and information present can be downloaded freely by the users. In addition to the development of MYCbase, analysis of compiled data provided valuable insights regarding the role of Myc in cancer. An example of a case study using breast cancer incorporating the application of MYCbase has also been described.

## Availability of data and materials

Database homepage: http://bicresources.jcbose.ac.in/ssaha4/mycbase. These data are freely available without restrictions for use by academics.

## Abbreviations

AP-MS: Affinity purification followed by mass spectrometry
BET: Bromodomains and extra-terminal motif proteins
bHLH-LZ: basic helix-loop-helix and leucine zipper region
ChIP-seq: Chromatin Immunoprecipitation followed by sequencing
CTD: C-Terminal domain
GO: Gene ontology
HATs: Histone acetyltransferases
MbI: Myc box I
MbII: Myc box II
NTD: N-terminal domain
PPIMs: Protein-protein interaction modulators
PPIs: Protein-protein interactions
PRPS2: Phosphoribosyl-pyrophosphate synthetase 2
PTM: Post-translational modification
S62: Serine 62
T58: Threonine 58
TF: Transcription factor.
